# Population decrease of *Scirpophaga incertulas* Walker (Lepidoptera Pyralidae) under climate warming

**DOI:** 10.1002/ece3.69

**Published:** 2012-01

**Authors:** Peijian Shi, Ling Zhong, Hardev S Sandhu, Feng Ge, Xiaoming Xu, Wei Chen

**Affiliations:** 1 State Key Laboratory of Integrated Management of Pest Insects and Rodents, Institute of Zoology, Chinese Academy of SciencesBeijing, China; 2 Plant Protection & Quarantine Bureau of Jiangxi ProvinceNanchang, China; 3 Everglades Research and Education Center, Institute of Food and Agricultural Services, University of FloridaBelle Glade, USA

**Keywords:** Density dependence, Generalized additive models, Growth rate, Linear model, Minimum annual temperature

## Abstract

*Scirpophaga incertulas* Walker is an important agricultural pest in Asia. Only few studies are available on its long-term population dynamics under climate warming. In this study, we used the linear and generalized additive models (GAMs) to analyze the historical dataset of >50 years on this pest at Xinfeng County of Jiangxi Province, China. The main objective of this study was to explore the effects of density (delayed) dependence and minimum annual temperature (MAT), which indirectly reflects climate warming, on the population dynamics of this pest. We found that both density dependence and MAT have significant influence on the annual population growth rate. The GAMs had relatively better applicability to the dataset than the linear models. Nonparametric model provided satisfactory goodness-of-fit (*R*^2^ > 0.5). At Xinfeng County, the MAT had a significant effect on the annual population growth rate of *S. incertulas.* The annual population growth rate of *S. incertulas* decreased with increase in MAT. Therefore, *S. incertulas* population becomes smaller and smaller in Southern China due to climate warming. The current study has two contributions: (1) providing a suitable method for predicting the annual population growth rate of *S. incertulas*, and (2) demonstrating that climate warming could decrease the *S. incertulas* population.

## Introduction

Population dynamics is an important area of study in ecology. Many ecological theories are related to population dynamics. Population dynamics of one species is affected by many factors. Previous studies showed the influence of density dependence on the insect population dynamics (Turchin 1990; Turchin et al. 1991; Friedenberg et al. 2008). Turchin (1990) built a single species population modeling of time-delayed density dependence to analyze the population dynamics of 14 forest insects, and found that eight cases exhibited clear evidence for delayed density dependence and log-induced oscillations. Crone (1997) extended this population modeling of time-delayed density dependence from a single species population dynamics to two interacting species population dynamics. The minimum annual temperature (MAT, or minimum winter temperature) was considered as the crucial factor limiting the northern distributions of many insects (Uvarov 1931; Ungerer et al. 1999; Tran et al. 2007; Shi et al. 2012). Friedenberg et al. (2008) combined the MAT with time-delayed density dependence in the single species population modeling. Previous study showed that the MATs in the Northern Hemisphere are increasing with time (Tran et al. 2007). Since the MAT has been demonstrated to have an important influence on distributions of insects, we are concerned of its influence on the annual population growth rate of insects.

**Figure 1:**
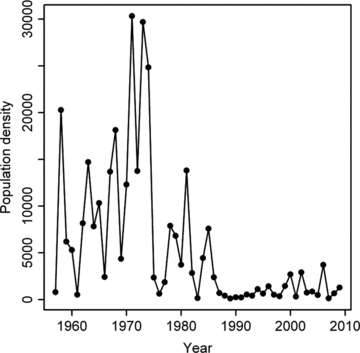
The population density of *S. incertulas* at Xinfeng County of Jiangxi Province, China.

*Scirpophaga incertulas* Walker is an important agricultural pest in Asia. It is always a traditional pest on rice in Southern China. Jiangxi Province of Southern China is contributing approximately 10% of the national total output of China. The damage of *S. incertulas* in Jiangxi Province has been a concern since long time (Zhong et al. 2000). In this study, the historical dataset of > 50 years (from 1957 to 2009) on *S. incertulas* collected from Xinfeng County of Jiangxi Province (Fig. 1) was used to determine the effects of density dependence and MAT on the population dynamics of *S. incertulas.* We also used this dataset to explore whether the delayed density dependence has a significant influence on the population dynamics of this pest.

## Materials and Methods

### Population density data

The data monitoring site (25°24.392′N, 114°50.008′E) was located in Xinfeng County, which has a typical subtropical climate. Based on the climate data of Xinfeng County Climate Station from 1986 to 2008, the annual mean temperature was 19.6°C, and the annual precipitation was 1492.1 mm.

In light trap, a 200 W incandescent lamp (19:00–0:00 h) was used from 1957 to 2005, which was replaced with 25 W helium lamp (18:00–6:00 h) in 2006. Each year, the monitoring began on March 1, and ended on October 31. No adults of this pest were found beyond this monitoring period in the past 53 years. We used the accumulative observed number of *S. incertulas* moths during monitoring period as the annual population density. The quotient of the population densities of two adjacent years was used as the annual population growth rate.

### Modeling

Turchin (1990) used the following equation to describe the insect population dynamics:


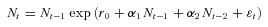
(1)

Here, *N_t_* is the population density at time *t*; 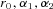 are constant; ɛ_*t*_ is a ra ndom error at time *t*. This equation can be described as:


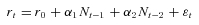
(2)

Here, *r_t_* = ln (*N_t_*/*N*_*t*−1_), which represents the annual population growth rate of population at time *t*. In general, the variable of *N*_*t*−2_ was added to significantly reduce the unexplained deviation relative to *r_t_* = *r*_0_+α_1_*N*_*t*−1_+ɛ_*t*_ (Turchin 1990; Turchin et al. 1991; Friedenberg et al. 2008). Sometimes *r_t_* = *r*_0_+α_1_*N*_*t*−1_+ɛ_*t*_ by itself can have a good fit to the data (Colchero et al. 2009). Equation 2 can be potentially modified if we relax the restriction of the linear relationship between the annual population growth rate and density dependence (Friedenberg et al. 2008).


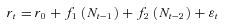
(3)

Here, *f_j_*(·) (*j* = 1, 2) is a specified smooth function. In practice, we can use the generalized additive models (GAMs; Hastie and Tibshirani 1986, 1990) to fit the data.

If there are some climatic factors with potential influence on the population dynamics, we can add these factors to equations 2 and 3:


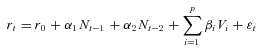
(4)


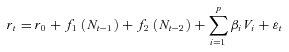
(5)

Here, *V_i_* represents the climatic factors, such as MAT (Friedenberg et al. 2008) and average summer standardized precipitation index value at time *t* that can reflect the extent of drought (Colchero et al. 2009); β_*i*_ are constant. We can modify equation 5 to:


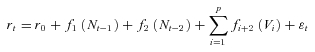
(6)

or we can further add some interaction terms among these factors. In general, we use equation 5 instead of equation 6 because the former can be easily explained. The former is a semiparametric model (one between a parametric model and a nonparametric model), but the latter is a nonparametric model. To determine the effect of MAT on the population dynamics, we could choose a semiparametric model that estimates the coefficient of MAT. If climate warming hypothesis held, the MAT would have a trend of increase. Hence, we used a simple linear model (Tran et al. 2007) to test whether there is a trend of increase for the MAT at Xinfeng County during these years.

Turchin's model and the following modified model in fact imply a hypothesis that *N_t_*_-2_ can significantly affect the annual population growth rate. This hypothesis was supported by the study of Turchin (1990). Here, we used the autocorrelation function (ACF) and partial autocorrelation function (PACF) to analyze whether the delayed density dependence could have influence on the annual population growth rate. If *N_t_*_-2_ significantly affects the annual population growth rate, we can follow equations 2–6; if not, we can delete the delayed density dependence. It is necessary to point out that ACF and PACF are generally restricted in the following model:


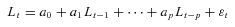
(7)

Here, *L_t_* = ln (*N_t_*).

## Results

### No delayed density dependence effect was found

**Figure 2:**
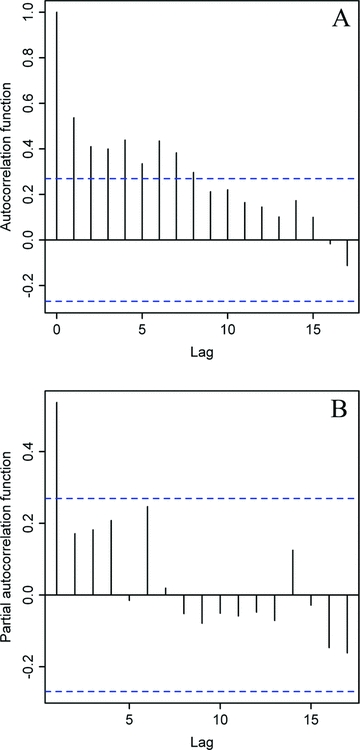
The autocorrelation and partial autocorrelation functions of the natural logarithm of *S. incertulas* population density. (A) Autocorrelation function. (B) Partial autocorrelation function. The dashed lines represent the 95% confidence interval.

The estimated ACF of 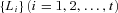 shows that there are autocorrelations in the time series; and the estimated PACF suggests the dropping of *L*_*t*−2_ from all the aforementioned equations (Fig. 2). There is no delayed density dependence effect except *N_t-_*_1_ that affects the dataset. Thus, we will not consider *N_t-_*_2_ in equations 2–6 in the following analyses.

### Fitted results by using the linear models

Table 1 shows the fitted results by using the linear models (equations 2 and 4). The *N_t_*_-1_ has a significant influence on the annual population growth rate (*P* = 0.0163 < 0.05 for the linear model without MAT; *P* = 0.0093 < 0.05 for the linear model with MAT). Addition of MAT did not improve the goodness-of-fit significantly. And the effect of MAT on the annual population growth rate is not significant (*P* = 0.0563 > 0.05). Intercept (*r*_0_) is not significant for the linear models with and without MAT. The linear relationship with MAT (*F*_2, 49_ = 5.174, and *P* = 0.0092 < 0.05) was slightly better than without MAT (*F*_1, 50_ = 6.176, and *P* = 0.0163 < 0.05), because the goodness-of-fit of the former is higher than that of the latter (whether *R*^2^ or *R*_adj_^2^). However, the goodness-of-fit is still too small, which indicates that equations 2 and 4 are not good models for the dataset. In nature, the nonlinear phenomena might be more common than the linear phenomena.

**Table 1:** Fitted results for linear models

Model	Parameter	Estimate	Standard error	*t* value	*p* value	*R^2^*	*R_adj_^2^*
Without MAT	Intercept	3.837e-01	2.488e-01	1.542	0.1294	0.1099	0.0921
	*N_t-_*_1_	−6.518e-05	2.623e-05	−2.485	0.0163		
With MAT	Intercept	−2.522e-01	4.054e-01	−0.622	0.5367	0.1744	0.1407
	*N_t–_*_1_	−6.937e-05	2.561e-05	−2.709	0.0093		
	MAT	−3.312e-01	1.694e-01	−1.955	0.0563		

Here, “e-0*x*” represents “×10^–*x*^”; *R^2^* represents the coefficient of determination; *R_adj_^2^* represents the adjusted coefficient of determination; MAT represents the minimum annual temperature.

### Fitted results by using the generalized additive models (GAMs)

**Figure 3:**
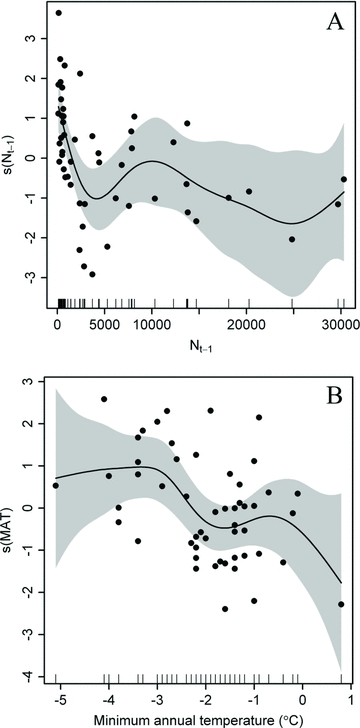
The additive nonparametric fit of the annual growth rate. The shaded region represents twice the pointwise standard errors of the estimated curve; the points represent partial residuals. (A) Partial residuals of the first variable, *N_t_*_-1_. (B) Partial residuals of the second variable, MAT.

Table 2 shows the fitted results by using the GAMs (equations 3, 5, and 6). The *N_t_*_-1_ is significant for these three models. The intercept is significant for the semiparametric model, and not significant for the other models. MAT is significant for semiparametric (*P* = 0.0041 < 0.05) model as well as nonparametric (*P* = 0.0384 < 0.05) model. It demonstrates that MAT can significantly affect the annual population growth rate. The coefficient of determination was 0.4380 for the semiparametric model and 0.5196 for the nonparametric model. This study shows that both density dependence and MAT had important influence on the annual population growth rate of *S. incertulas* (Fig. 3). The latter had a negative influence on annual population growth rate.

**Table 2:** Fitted results for generalized additive models (GAMs)

Model	Item	Degrees of freedom	Estimate	Standard error	*t* value	*p* value	*R^2^*	*R_adj_^2^*
Without MAT	Intercept		0.0094	0.1833	0.051	0.959	0.2899	0.223
	*s*(*N_t–_*_1_)	4.4112				0.0148		
With MAT (semi-parametric)	Intercept		–0.9124	0.3472	–2.628	0.0117	0.4380	0.358
	*s*(*N_t–_*_1_)	5.3268				0.0013		
	MAT		–0.4627	0.1529	–3.025	0.0041		
With MAT (nonparametric)	Intercept		0.0094	0.1604	0.059	0.954	0.5196	0.404
	*s*(*N_t–_*_1_)	5.2786				0.0025		
	*s*(MAT)	4.5994				0.0384		

### Evidence of climate warming from the minimum annual temperature (MAT)

**Figure 4:**
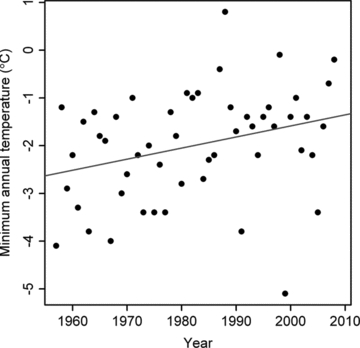
The linear relationship between the minimum annual temperature and time.

MAT increased linearly (*F*_1, 50_ = 5.132; *P* = 0.02785 < 0.05) with increase in time (Fig. 4). The slope was significant (*P* = 0.0279 < 0.05), which indicates that the increase in MAT at Xinfeng County was because of climate warming. In fact, using the linear model, Tran et al. (2007) also proved an increasing trend of MAT with time (1960–2004) in the southeastern United States.

## Discussion

Friedenberg et al. (2008) considered the interaction between (delayed) density dependence and climate variables. In equations 5–6, we use the GAMs with an important climatic variable (i.e., MAT) to describe the annual population growth rate. If there was an interaction between *N*_*t*−1_ and MAT, it would be necessary to define the interaction, such as α_3_·*N*_*t*−1_· MAT, where α_3_ is constant. In a GAM, the interaction needs a manual definition. However, in practice, we cannot clearly provide the detailed formulation of this interaction. In this study, we have neglected the interaction, but we found that the goodness-of-fit without an interaction was still good. In order to show the feasibility of neglecting the interaction, we consider whether the interaction can affect significantly the annual population growth rate in this section. Here, we replace equations 5–6 by the following equation:


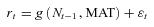
(8)

Here, *g* is the regression surface. We use the local regression models (LOESS or LOWESS, Cleveland et al. 1992) to replace the GAMs. For the local regression models, there is no explicit specification that rules out interactions (Cleveland et al. 1992). Thus, we do not need to know the detailed formulation of the interaction, because this interaction is naturally integrated into the local regression models. By setting the smoothing parameter to 0.5, we estimated *R*^2^ = 0.5502 which was slightly greater than the estimation (*R*^2^ = 0.5196) through nonparametric model (Table 2). It implies that we could neglect interaction between density dependence (*N_t_*_-1_) and MAT. When we added an interaction of *N*_*t*−1_· MAT in the nonparametric model (i.e., equation 6), it was not significant (*P* = 0.6575 > 0.05). Yamamura et al. (2006) used LOWESS to analyze the population dynamics of three agricultural pests. However, equation 5 published in their paper has a serious problem. They claimed that “LOWESS is additive,” which was used to derive equation 5 published in their paper, but in fact this precondition does not hold (see Cleveland et al. [ 1992] for details).

**Figure 5:**
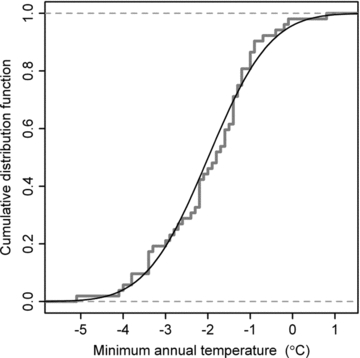
Relationship between cumulative distribution function and MAT. The black line represents the normal accumulative distribution function; and the grey line represents the empirical distribution function. *F*(*MAT* < −11) ≍ 0, indicates that the probability of reaching lower lethal temperature at Xinfeng County approximates 0.

**Figure 6:**
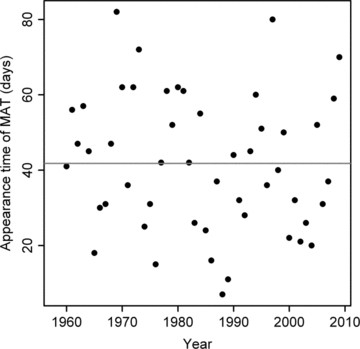
The appearance time of MAT during different years of study. The grey line represents a constant of 41.78th day of each year, which is January 11.

Kiritani (1988) reported a descending trend of annual changes in area under paddy (in hectares) infested by *S. incertulas* from 1937 to 1978 in Japan. At present, it is rather difficult to find the wild population of this pest in Japan (personal communication with Dr. Ikemoto Takaya). We speculate that this phenomenon might partially be triggered by the increasing MATs in Japan. The previous studies related to the effects of global warming on insects were usually concentrated on two aspects: (1) movement northward of distribution limits, and (2) population density change. Those studies showed that global warming lead to the increasing damage of pests (e.g., Logan et al. 2003; Kiritani 2006; Diffenbaugh et al. 2008). However, our study shows that global warming lowered the population of *S. incertulas* at Xinfeng County. This pest is in fact very sensitive to temperature, and its volitism can change when exposed to different climate environments (Zhang 1992; Stevenson et al. 2005). Under global warming, the number of generations per year could increase, but the increasing number of generations will not aggravate the damage by this pest in Southern China (Shi et al. 2012). In Southern China, such as Jiangxi Province, MAT is less than the lower lethal temperature of *S. incertulas*. The lower lethal temperature could be approximated by supercooling point (Ungerer et al. 1999). Based on the study of Zhang (1990), this lower lethal temperature is about –11°C. From our >50 years MAT data, the probability of reaching the lower lethal temperature could be calculated on the condition of MAT ∼ iid(–1.99, 1.15). The MAT data at Xinfeng County passed the Shapiro–Wilk test (*W* = 0.9782, *P* = 0.4529 > 0.05; Xue and Chen 2007), which means that the null hypothesis of MAT normality could not be rejected. The probability of reaching the lower lethal temperature approximates zero (Fig. 5). In other words, MAT has little influence on the winter mortality of population. In addition, the appearance time of MAT at Xinfeng County is rather stable. We used December 1 of the former year as the start day (i.e., 0), and calculated the appearance time of MAT of the next year in days. We performed linear regression on the appearance time versus time (Fig. 6). The slope was not significant (*P* = 0.404 > 0.05). Then we performed linear regression without slope, and obtained intercept = 41.78 (*P* < 0.05). Thus, the appearance time of MAT in Xinfeng County is estimated to be January 11. It is necessary to point out that MAT is increasing with time although its appearance time is approximately constant. Consequently, we speculate that increasing MAT could result in the advances of the biological time of rice and pest development. However, the advance of the biological time of rice development might be different from pest development. This pest could not synchronize its development with rice, which might lead to the population decrease of *S. incertulas*. It deserves further study.

In summary, the GAMs can provide an approximate description for the population dynamics dataset of *S. incertulas* at Xinfeng County. A delayed density dependence has no influence on the annual population growth rate. Both density dependence and MAT have important influence on the population annual growth rate of *S. incertulas* at Xinfeng County, but there was no interaction effect.
